# Perceived malaria in the population of an urban setting: a skipped reality in Dakar, Senegal

**DOI:** 10.1186/1475-2875-11-340

**Published:** 2012-10-08

**Authors:** Abdoulaye Diallo, Stéphanie Dos Santos, Richard Lalou, Jean-Yves Le Hesran

**Affiliations:** 1 Institut de Recherche pour le Développement, UMR 216, Mère et enfant face aux infections tropicales, 4 avenue de l’Observatoire, Paris, 75270 cedex 06, France; 2 Université Paris Descartes, Sorbonne Paris Cité, Faculté des sciences pharmaceutiques, 4 avenue de l’Observatoire, Paris, 75270 cedex 06, France; 3 Institut de Recherche pour le Développement, UMR151, Laboratoire, Populations, Environnement et Développement, Dakar, Sénégal; 4 Institut de Recherche pour le Développement, UMR151, Laboratoire, Populations, Environnement et Développement, Marseille, France

## Abstract

**Background:**

Urban malaria remains a public health problem. Dakar is located in a low endemic area. However, anti-malarial drugs consumption is reported to be high despite the decline of malaria announced by health authorities. The objective of the present study was to assess the burden of reported malaria attacks (RMAs) in 2008 and to describe care-seeking behaviours in the population of Dakar, Senegal.

**Methods:**

In this cross-sectional study, 2,952 households selected from 50 sites were visited. In each household, a women and a child between two and 10 years old were interviewed about a malaria episode that occurred in 2008. The following information was recorded: age, education level, sex (for children), type of care seeking, method of diagnosis, use of anti-malarial treatment, place of medication purchase, bed net use, malaria-related deaths in the family, and perceptions of the frequency of mosquito bites. After a description of the variables in each subsample, a Pearson’s chi-square test was used to compare proportions, and logistic regression was performed to identify the association between RMAs and other covariates.

**Results:**

Among women, 31.8% reported a malaria attack in 2008; among children, the rate of malaria attacks reported by mothers or caretakers was 39.0%. With regard to care-seeking, 79.5% of women and 81.5% of children with a RMA had visited health facilities (HFs). Younger women and children under five years old were more likely to visit a HF (P<0.001). Presumptive diagnosis was the primary method that was used to identify malaria in HFs. For those who had visited a HF, the rate of anti-malarial treatment was 77% in women and 60% in children. Finally, 43.6% of women and 42.0% of children declared the use of bed nets. In a multivariate analysis, the malaria-related death of a relative and perceptions of mosquito bites were significantly associated with RMAs in women. In children, age was associated with RMAs.

**Conclusion:**

The frequent perceptions of the occurrence of malaria in the population were confirmed at the HF by the high presumptive diagnosis of health professionals. Despite the decline of malaria that has been announced by health authorities, the population will continue to complain of malaria and seek care directly at private pharmacies. This situation may sustain the circulation of anti-malarial drugs and increase the risk of an emergence of anti-malarial resistance.

## Background

Malaria is reported to be more prevalent in rural areas compared to urban settings [1,2]. It is well known that urbanisation is highly associated with the decline of malaria transmission [3]. Indeed, many studies have reported little or no malaria transmission in major African cities, as urban settings are less favourable for vector breeding sites [4,5]. However, despite a low rate of transmission, malaria remains a major public health problem in urban areas, as it currently impacts more than 50% of the African population and is expected to impact at least 60% of the population by 2050 [6]. Moreover, *Anopheles* mosquitoes can potentially breed in polluted urban areas [7,8], which suggests the possibility of an increase in the transmission of malaria.

Dakar area is located in a low transmission area. First two studies in suburbs (Pikine) [9,10] had found in the early 80’s an annual entomological inoculation (EIR) rate was 47 in 1979 and 0.4 in 1988. In addition parasite prevalence was 8.8% in children aged 6 months to 6 years [9] and 3.8% in all-age population [10].

Others studies have described malaria transmission in Dakar. In the south and central sanitary districts in 1994–95 and 1996–97, respectively [11,12], *Anopheles arabiensis* aggressiveness was low, and no infected *Anopheles* was collected. In the central area, a parasite prevalence of 1% and an annual incidence of clinical attacks of 2.4% have been recorded. However, during 2005–2006, malaria transmission was assessed in two vegetated areas of downtown Dakar; annual EIRs were 9.5 and 4, respectively, in Bel-air and 3 and 3, respectively, in Ouakam [13].

Despite the low malaria endemicity that was previously reported in Dakar, one study has emphasized the high level of anti-malarial drug consumption for fever [14]. In addition, self-medication with anti-malarial drugs is common in the general population [15,16]. Recent data from the Senegal Ministry of Health have shown a decline in malaria. Morbidity in public health facilities decreased from 17.9% in 2007 to 2.6% in 2008 at Dakar [17]; indeed, 2006–2007 period saw the implementation of artemisinin-based combination therapy (ACT) and rapid diagnostic tests (RDTs) in malaria case management. However, these data are biased, as they consider only public facilities and do not consider data from the private health system. Moreover, these data rely exclusively on RDT results, which have been achieved at only a rate of 52%. Because self-medication is common, an important segment of the population does not visit health services when the people experience episodes of malaria. Therefore, this segment is not recorded in health statistics and constitutes a loss of useful information. This information is needed for assessing the malaria control programmes and for planning future health actions.

In 2008, a three-year, multi-disciplinary research programme was undertaken in Dakar to identify the determinant factors of health care access and the success of new strategies (i e, RDTs and ACT) in malaria control at the population level. The aim of the present work was to assess the burden of malaria attacks reported by approximately 3,000 households in the Dakar area.

## Methods

### Study area

The study took place in the region of Dakar, Senegal, which comprises the districts of Dakar, Pikine, Guediawaye and Rufisque. In 2002, these four urban districts had a population of 2,168,314 inhabitants and 302,551 households [18]. Based on a 2.5% annual growth rate, the population in the Dakar region was estimated to be 2,493,561 in 2008. Housing types are residential, planned, “spontaneous” legal and “spontaneous” illegal. In the region of Dakar, spontaneous housing represents more than 30% of inhabited areas. The rate of illegal housing is estimated to be 21.7% in the entire region, with 2.9% for the district of Dakar, 42.4% for Pikine and 9.5% for Rufisque [19].

The region of Dakar is in a Sudano-Sahelian climate with a long dry season (October to June) and a short rainy season (July to September). The level of recorded rainfall rarely exceeds 500 mm per annum. However, it has been increasing since 2005 reaching 510 mm in 2008; most of this quantity fell in one week, causing major floods in the suburban areas of Dakar [20]. Due to the proximity of the sea, this coastal area benefits from particularly mild conditions, with a maximum temperature of 27-28°C in September and a minimum of 21-22°C in February.

### Study type and population

A cross-sectional survey was performed from October to December 2008. Following the model of household surveys using the Grouped Islets for Statistic Indicators (IRIS) carried out within the SIRS research programme (health, inequalities and social breakdown in France) [21], 3,000 households were to be visited. The eligible households must contain at least one child aged between two and 10 years.

### Selection of study sites

The detail of the procedures for the selection of study sites was described in a first paper [22].

The urban environment is known to be a heterogeneous space from both a socio-economic and an environmental perspective. Briefly, the aim of the choice of study sites was therefore to highlight the diversity of the urban zone. It was necessary to have homogeneous zones and the most heterogeneous zones between them.

### Selection of the households and the individuals

3000 households selected in 50 sites were to be visited. The field worker picked up a concession within the study zone and moved from one household to the next following sampling procedure. If a concession encompassed several households, the field worker chose the head of the household using the first name ranked alphabetically. Sixty households in each site (3,000 households for the 50 sites) were to be selected. The first criterion for household selection was the presence of at least one two to 10-year-old child. After obtaining family agreement, socio-demographic investigators completed a questionnaire about the household lifestyle, education level, income, and the access mode to healthcare facilities. A second questionnaire was addressed to an adult woman (generally the child’s mother) and a two to 10-year-old child. The questionnaire asked the respondents if they had experienced a malaria attack in 2008. For reported malaria attacks (RMAs), the questionnaire explored the following related events: type of care seeking, method of diagnosis, place of diagnosis, treatment received, and place of medication purchase. Other information was collected, including deaths of relatives perceived or confirmed to have been caused by malaria, preventive measures (the use of a bed net), and perception of the frequency of mosquito bites.

### Ethics statement

This protocol was approved by the Ethics Committee of Senegal’s Department for Health (SEN 12/08). Information about the objective of the study was given to the head of the household, and verbal consent was requested. For minors, the consent of their legal guardians was requested. This procedure was used because most heads of household could not write. A nurse explained the aims and interest of the study, and then signed a document proving that consent was obtained.

### Statistical analysis

The statistical analysis was performed using Stata version 11 (Stata Corp., Texas, USA, 2009). The socio-demographic characteristics (age, sex) of the respondents were described. A Pearson’s chi-square test was used to compare proportions. To estimate the association between a RMA and the covariates, a logistic regression analysis was performed in the two groups. The following covariates were considered: age, education level (for women), use of a bed net, sex (for children), malaria-related deaths in the family, and perceptions of the frequency of mosquito bites. Women were divided into four age classes based on quartiles. In children, two age classes were created (<5 years and ≥5 years). A test was considered to be statistically significant if the p-value was less than 0.05.

## Results

### Prevalence of reported malaria attacks in two groups by study sites

The analysis included 2,451 women and 2,380 children who were selected from 49 sites in the Dakar area. One site (Point E) was not included in the analysis because of an insufficient number of households. The median age was 34 years (range = 15–77) for women and five years (range = 2–10) for children. Among women, 32% reported a malaria attack in 2008; among children, the rate of malaria attacks that was reported by mothers or caretakers was 39% (Table 1).

**Table 1:** Malaria care-seeking behaviour in women and children, Dakar, 2008

**Covariates**	**Women (2,451)**	**Children (2,380)**
**Reported malaria attacks in 2008**	31.8% (780/2,451)	39% (930/2,380)
**Hospital care-seeking**		
** Yes**	79.5% (618/777)	81.5% (750/920)
** No**	20.5% (159/777)	18.5% (170/920)
**Method of diagnosis**	N=558	N=816
** Microscopy**	6.8%	1.2%
RDT*	8.2%	4.6%
** Presumptive**	70.6%	80.1%
DNK**	14.3%	14%
**Received treatment (Yes)**	92.4% (720/779)	95.1% (881/925)
**Used anti-malarial drugs (Yes)**	68.8% (496/720)	55.7% (492/884)
**Place of medication purchase (anti-malarials)**	N=486	N=477
** Health services**	49.8%	47%
** Official drugstores**	48.7%	51.4%
** Shops, market, gift**	1.44%	1.7%
**Self-medication by an anti-malarial**	4.5%	
**Self-medication by another treatment**	13.5% (94/720)	
**Use of a bed net (Yes)**	44.8% (1,093/2,438)	42.4% (1,009/2,380)
**Death of a relative (Yes)**	9.6% (233/2,431)	

DNK**: Do not know.

RDT*: rapid diagnostic test.

N: sample size.

**Figure 1:**
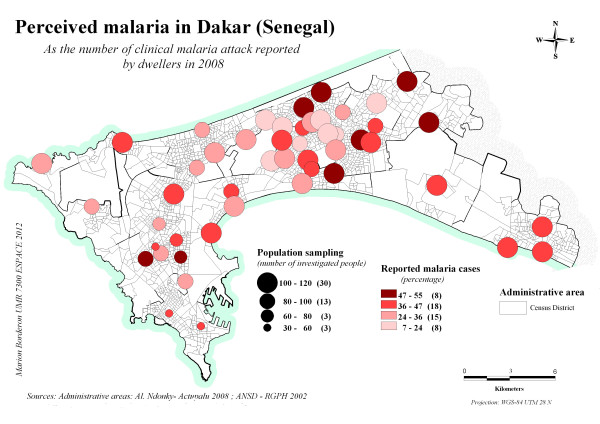
Rate of reported malaria attacks (by women and children) by study sites, Dakar, 2008.

The rate of RMAs by sites varied between 10% (Maka colobane 1) and 50% (Biscuiterie) in women, and the rate reached as high as 65% (Wakhinane, Baobab, Alioune Sene) in children (Figure 1). In every site, the RMA rate reached at least 10%, which suggests that perceptions of the presence of malaria are widely distributed throughout Dakar.

**Figure 2:**
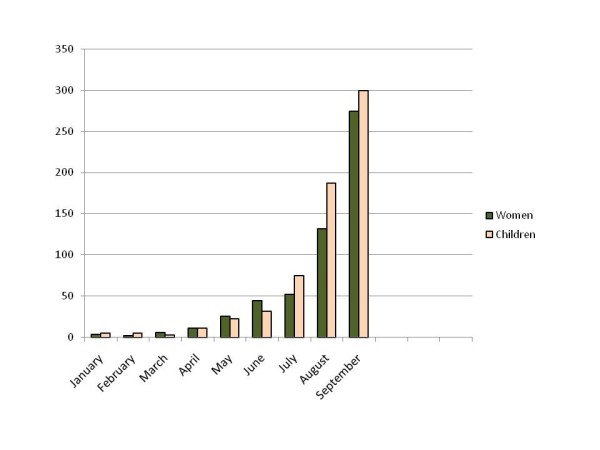
Number of monthly malaria attacks reported by residents (women and children), Dakar, 2008.

High rates of RMA (> 45%) were found in sites near the city centre and in the suburbs near the marshland (Niayes). In the Niayes area, the permanent humidity allows for gardening activities throughout the year and creates potential breeding sites. The number of RMAs increased gradually in the two groups from April to September, which is at the end of the rainy season (Figure 2).

### Use of health facilities

A total of 79.5% of women and 81.5% of children with a RMA had visited a health facility (HF). Younger women were more likely to have visited a HF (trend test for equal odds, P < 0.001). Mothers or caretakers of children under five years old were more likely to have referred their children to a HF (P <0.001). Similarly, in children, males were more likely to have visited a HF (P = 0.009).

In HFs, presumptive diagnosis was used to identify malaria in 70.6% of women with RMAs. Only 15.0% of women underwent a blood test (thick smear, or RDT); however, 14.0% of women did not remember if they had had a blood test. In children, 80.0% had undergone a presumptive diagnosis, and approximately 5.8% had had a blood test; 40 (14.0%) respondents were unable to provide information about the diagnostic method.

### Treatment

When visiting a HF, 76.7% (459/598) of women with a RMA had received an anti-malarial treatment (AT). Among women who had not visited a HF, 28.2% (35/123) had taken an AT (P <0.001); 22.4% (36/159) had not taken any treatment. The remaining women had received antipyretic treatment (primarily paracetamol). Four women had received a traditional treatment.

A total of 13.0% (94/720) of women with a RMA declared having self-medicated. Among children who had visited a HF, 60.4% had received an AT; among children who had not visited a HF, 34.4 % had received an AT. Approximately 48.7% of women had purchased an AT in a private pharmacy, while 49.8% of women had purchased an AT in a HF. Only 1.44% of women reported having purchased an AT in a market or shop.

### Preventive measures

Bed nets were reportedly used by 44.8% of women and 42.4% of children. Surprisingly, there was no association between a RMA and bed net use in women (P = 0.346) or children (P = 0.716).

### Risk factors of reporting malaria attacks in 2008

In women, malaria-related death in the family was significantly associated with a RMA (Table 2) in the univariate and multivariate analysis (Odds ratio = 1.46, P = 0.012). Perception of the frequency of mosquito bites was significantly associated with a RMA. Individuals who reported frequent bites were more likely to have a RMA (trend test for equal odds, P= 0.003) compared to individuals who reported few or no bites. Children under five years old (Table 3) were more likely than older children to have a RMA (Odds ratio = 0.76, P = 0.001).

**Table 2:** Association between reporting a malaria attack and covariates in women (logistic regression)

			**Univariate analysis**		**Multivariate analysis**	
**Variables**	**Class**	**N° of malaria attack**	**OR (95%IC)**	**P**	**OR (95%IC)**	**P**
**Age**						
	<= 27	216	1	0.252	1	0.267
	> 27 et <= 34	199	0.81(0.64-1.03)		0.78(0.61-1.01)	
	> 34 et <= 41	176	0.86(0.67-1.10)		0.90(0.69-1.16)	
	> 41	189	0.80(0.63-1.02)		0.81(0.63-1.06)	
**Education level**						
	None	310	1	0.057	1	0.060
	Primary school	178	1.02(0.83-1.24)		1.01(0.83-1.24)	
	High school	113	0.75(0.58-0.97)		0.75(0.57-0.97)	
**Death of a relative**						
	No	676	1	0.001	1	
	Yes	96	1.57(1.19-2.07)		1.46(1.08-1.96)	0.012
**Use of a bed net**						
	No	440	1	0.346	**	**
	Yes	338	0.92(0.77-1.09)			
**Mosquito bites**						
	None	44	1	0.003*	1	0.009
	Few	235	1.66(1.15-2.41)		1.65(1.10-2.46)	
	Many	499	1.81(1.28-2.58)		1.80(1.23-2.65)	

**: covariate not introduced in the multivariate analysis.

*: trend test for odds.

N°: number.

P: p-value.

**Table 3:** Association between reporting a malaria attack and covariates in children (logistic regression)

			**Univariate analysis**		**Multivariate analysis**	
**Variables**	**Class**	**N° of malaria attack**	**OR(95%IC)**	**P**	**OR(95%IC)**	**P**
**Age**						
	<= 5	421	1	0.001	1	0.001
	> 5	509	0.76(0.64-0.90)		0.76(0.64-0.89)	
**Sex**						
	Male	440	1	0.450	1	
	Female	490	1.06(0.90-1.25)		1.07(0.91-1.26)	0.387
**Use of a bed net**						
	No	540	1	0.716	**	**
	Yes	390	0.96(0.82-1.14)			

**: covariate not introduced in the multivariate analysis.

N : number.

P : p-value.

## Discussion

The aim of the present study was to assess the malaria burden as it is experienced by urban dwellers. The data showed that malaria was perceived as a common disease, as one third of surveyed individuals had a RMA in 2008, and most of the respondents reported a RMA after the beginning of the rainy season. This study was primarily focused on women and children between two and 10 years old. Women were more appropriate than men to interview as women are responsible for the daily activities in the home. Men are often absent because of work or travel. Therefore, women were more likely to provide relevant information about children’s febrile illnesses.

The data that were collected in this study are declarative. However, the aim of the study was not to measure the prevalence of malaria; the aim was to obtain information about perceptions and behaviours concerning malaria in the population. The interviews were performed after the rainy season. This approach allowed avoiding memory bias in responses, as most malaria attacks occur during the rainy season. However, approximately 20% of the dwellers reported malaria attacks that occurred in the dry season, which showed that health professionals and the population could considered a febrile episode as a malaria attack at any time of the year.

To date, microscopy has been the standard method to measure *Plasmodium* prevalence in the population. In Dakar, the prevalence in the population was reported to be low [12], which suggests a low burden of malaria attacks. This observation was not confirmed by respondents in this study, who exhibited RMAs greater than 30%. However, in 2008, *Plasmodium* carriage was measured by PCR in Dakar [22] and *Plasmodium* prevalence, at approximately 16%, was found to be relatively high. However it is not possible to make any link between the rate of *Plasmodium* carriage and the rate of RMAs since presumptive diagnosis was used at health facilities. In addition data collected in an other survey by the same team (unpublished data) showed that malaria is commonly diagnosed among febrile patients in HFs. However these findings should be confirmed by a large study of morbidity using active screening of malaria cases.

The results also showed that presumptive diagnosis is frequently used in HFs (73% for women and 80% for children), despite the implementation of RDTs in September 2007. In Dakar, the rate of presumptive diagnosis is similar to the rate that was described 20 years ago by Faye *et al.*[23]. The rate suggests an over-diagnosis of malaria attacks, which is consistent with two studies. Othnigué [24] found that only 30% of clinically diagnosed malaria cases were positive for *Plasmodium*, and in Thailand, Luxemburger [25] reported that using a clinical diagnosis would result in the prescription of an anti-malarial drug for 30% of non-malaria febrile episodes.

This over-diagnosis in HFs in Dakar could lead to a pernicious effect by stoking perceptions that malaria is common. As a consequence, malaria may be over-diagnosed in febrile patients, which was reported by Ndour [23] in rural Senegal. In this study, 61% of respondents reported fever to be a common symptom of malaria.

Two primary reasons could explain the difficulty of assessing the actual malaria burden in the population. First, most cases of malaria are presumptive. Second, self-medication is common, and self-medication is not reported in health statistics. As a consequence, anti-malarial consumption is enhanced by the high circulation of drugs that are available without prescription in private pharmacies or in informal markets.

In the present study, 80% of individuals had visited a HF. This rate is higher than the rate found by Franckel in a rural area in Senegal, where the use of HFs varied from 5 to 45% [26]. The use of HFs in cases of fever appeared to be a routine practise in the urban population.

The use of HFs decreased with age in women and children. For women, who are often caretakers of children, previous experience of malaria episodes or in HFs allows for self-diagnosis and self-medication, described by Baxerres and Le Hesran [27] in rural Senegal. Family history appears to play a major role in creating perceptions of malaria and could be a determinant of care-seeking behaviour.

In 2003, Ndiaye *et al.* found that 40% of 271 febrile patients had self-medicated before visiting a HF [15]. These data cannot be compared to those found in the present study. No differentiation was made between self-medication as the first intention and self-medication as an exclusive health-seeking behaviour. However, global self-medication appeared to be more important in the population. McCombie [28] reported that the self-purchasing of drugs ranged from 4 to 87%, and half of the cases relied exclusively on self-treatment, usually with anti-malarials.

Perceptions of the frequency of mosquito bites was significantly associated with a RMA; this association may be explained by the fact that individuals know that malaria is transmitted by mosquitoes [29]; therefore, the presence of more mosquitoes is associated with a greater perception of malaria. Another study conducted in pregnant women in Nigeria [30] showed that 78.9% of surveyed women identified mosquito bites as the cause of malaria. Even if the majority of mosquitoes are not *Anopheles*, malaria transmission is still possible, which has been shown by a study that was conducted with the same population. By measuring human antibodies against *Anopheles* saliva, the study showed that contact between humans and mosquitoes occurs frequently in the Dakar area [31].

Recent entomological data in Dakar [13,32] provide evidence of malaria transmission. In addition, *Plasmodium* carriage was found to be important [22]. These results suggest that a part of RMAs (unknown perhaps underestimated) could be actual cases of malaria attacks despite the high use of presumptive diagnosis.

The data showed a lack of evidence for heterogeneity in RMA prevalence between sites. Entomological studies and *Plasmodium* prevalence suggested variation in malaria transmission in Dakar. This discrepancy could be observed because each unexplained fever could be considered to be a malaria attack by individuals, even if there is no condition for malaria transmission in their environment.

Finally, there is a convergence of factors that provide evidence for a malaria risk in the population in Dakar: a high frequency of fever in the rainy season, malaria-related deaths, high rates of mosquito bites, permanent floods in many sites, and presumptive diagnosis in HFs. It is difficult to estimate the actual burden of malaria in the population, but it is clear that perceptions of the occurrence of malaria could persist.

## Conclusion

The frequency of perception of occurrence of malaria in the population was confirmed by visiting HFs, where a high presumptive diagnosis rate was reported by health professionals. This perception of malaria could sustain a high consumption of anti-malarial drugs.

Despite the decline of malaria that has been announced by health authorities, the population will continue to complain about malaria and seek care directly from private pharmacies. This situation may sustain the circulation of anti-malarial drugs and increase the risk of an emergence of anti-malarial resistance.

Further studies are necessary to differentiate perceived and real cases of malaria and to identify the actual burden of the disease in urban Dakar. Efforts are needed to sensitize health professionals to use confirmatory diagnostic tools more effectively before they prescribe anti-malarials and to urge the population to avoid seeking care outside HFs.

## Competing interests

The authors declare that they have no competing interests.

## Authors’ contributions

AD participated in study design, supervised field operations, carried out the acquisition, the statistical analysis and the interpretation of data and drafted the manuscript. SDS participated in the design and was responsible for the coordination of the project. RL coordinated the project and participated in the design of this study. JYL was responsible for the coordination of the study, participated in the design and supervised the writing of the manuscript. All authors read and approved the final manuscript.

## Editing

This manuscript was edited for English language by American Journal Expert (Editorial certificate No F707-33FC-272C-F956-7EB1).
